# The μ2 and λ1 Proteins of Mammalian Reovirus Modulate Early Events Leading to Induction of the Interferon Signaling Network

**DOI:** 10.3390/v14122638

**Published:** 2022-11-26

**Authors:** Guillaume David Després, Kenny Ngo, Guy Lemay

**Affiliations:** Département de Microbiologie, Infectiologie et Immunologie, Université de Montréal, Montréal, QC H2R 2V7, Canada

**Keywords:** reovirus, interferon, RIG-I, viral RNA, ISGs, Stat1

## Abstract

It has been previously shown that amino acid polymorphisms in reovirus proteins μ2 and λ1 are associated with differing levels of interferon induction. In the present study, viruses carrying these polymorphisms in either or both proteins, were further studied. The two viral determinants exert a synergistic effect on the control of β-interferon induction at the protein and mRNA level, with a concomitant increase in RIG-I. In contrast, levels of phospho-Stat1 and interferon-stimulated genes are increased in singly substituted viruses but with no further increase when both substitutions were present. This suggests that the viral determinants are acting during initial events of viral recognition. Accordingly, difference between viruses was reduced when infection was performed with partially uncoated virions (ISVPs) and transfection of RNA recovered from early-infected cells recapitulates the differences between viruses harboring the different polymorphisms. Altogether, the data are consistent with a redundant or complementary role of μ2 and λ1, affecting either early disassembly or the nature of the viral RNA in the incoming viral particle. Proteins involved in viral RNA synthesis are thus involved in this likely critical aspect of the ability of different reovirus variants to infect various cell types, and to discriminate between parental and transformed/cancer cells.

## 1. Introduction

Mammalian reovirus has been instrumental in our understanding of viral replication and interaction with either host cell or the infected organism [1,2,3,4]. More recently, in the last twenty years or so, reovirus has been under study as one of the many viruses exhibiting preferential tropism for cancer cells [5,6,7]. In addition to a gain of fundamental knowledge, it is thus of interest to examine the viral determinants of replication under different conditions, in order to better adapt the virus to various cancer cell types [5,6,7]. A major determinant of host-cell resistance to viral infection is the interferon response, it is especially significant in the context of viral oncolytic activity since cancer cells are often deficient in this response, although the impact could vary between cancer types [8,9,10,11,12,13].

Previous work has shown a much higher induction of type I interferon in the serotype 3 Dearing laboratory stock T3D^K^, recovered by plasmid-based reverse genetics from the currently used plasmids, compared to our laboratory stock, T3D^S^. Determination of genome sequence allowed to reconstruct the T3D^S^ virus by reverse genetics [14] and to establish that this phenotype was solely due to polymorphisms in the M1 and the L3 gene [15], respectively, encoding viral proteins μ2 and λ1, while both μ2 and λ2 are responsible for the level of sensitivity of the virus to the interferon response.

Despite its limited pathogenicity in humans, mammalian reovirus has been instrumental in our understanding of viral replication and pathogenesis. It was also shown to preferentially infect and destroy cancer cells, leading to various clinical trials [5,6]. However, reoviruses presenting an increased oncolytic activity or better oncotropism should be developed and it is currently envisioned that different virus variants will be required to efficiently and safely attack different cancer cell types [16,17]. From another point of view, there is accumulating evidence supporting the possibility of emerging pathogenic reoviruses, including from bats, in humans or domestic animals (for examples among many others: [18,19,20,21]). Induction, repression and level of resistance to the interferon response is well recognized as a key aspect of viral pathogenicity and emergence. It is also critical in the ability of many oncolytic viruses to discriminate between parental and cancer cells [8,12,13]. A better understanding of viral determinants in interferon induction or sensitivity requires an in-depth knowledge of viral and cellular determinants involved.

In the present study, our initial results were confirmed and we further showed that substitution of both genes of the low interferon-inducing virus by those of the high-inducing virus were required to completely recover the phenotype of interferon induction, both at the protein and mRNA level. Further study of gene expression along the interferon signaling network suggests that the viral determinants are acting at the level of initial events of viral recognition during the interferon response. This is also consistent with the observation that co-infection with interferon inducing and non-inducing viruses results in intermediate levels of induction, and that difference between viruses was abolished when infection was performed with partially uncoated virions (ISVPs). Furthermore, while transfection of RNA from late-infected cells resulted in similar induction of interferon independently of the virus used, the induction with RNA from early-infected cells was higher and differs between viruses. This suggests, again, that the effect is exerted early during infection. This is likely the consequence of an effect of the two proteins on either viral disassembly or on the packaging or structure of viral genomic RNA.

## 2. Materials and Methods

### 2.1. Cell Lines and Viruses

L929 mouse fibroblasts were originally obtained from the American type culture collection and were grown in minimal Eagle medium (MEM) with 5% fetal bovine serum. The wild-type virus from Terence Dermody’s laboratory stock was recovered from the original plasmids currently used in reverse genetics [22] and will be referred to as T3D^K^ (for Kobayashi). The laboratory stock from our laboratory, referred to as T3D^S^ (for Sandekian), was reconstituted by site-directed mutagenesis of the original reverse genetics plasmids, and was previously described [14]. Viruses harboring either the T3D^K^ μ2-encoding M1 gene, λ1-encoding L3 gene or both in the T3D^S^ background were rescued by reverse genetics, and were previously described [15]. In the different figures, “none” refers to the T3D^S^ virus with no substituted gene, thus encoding both μ2 and λ1 from T3D^S^. All virus stocks were routinely propagated on monolayers of L929 cells. Dishes with infected cells and its medium were submitted to three cycles of freeze (−80 °C) and thaw (room temperature) approximately 24 h post-infection to prepare infectious virus stocks.

Viral stocks were kept in aliquots at −80 °C and were titrated before use. Virus titers were routinely determined by TCID_50_ on L929 cells [23]. All titers were determined in triplicates and average value obtained was then used in all experiments. Virus stocks for all 4 viruses under study had viral titers that differ by less than 6.5-fold and the difference between the 4 viruses under study was not statistically significant.

Infectious subviral particles (ISVPs) were prepared from infectious viral stocks as previously described [24]. Briefly, viral stocks were prepared in absence of serum and treated with 20 μg/mL of chymotrypsin for 30 min at 37 °C, control viral stocks were similarly incubated but in the absence of chymotrypsin. Proteins were analyzed by immunoblotting in order to verify cleavage of μ1C to δ, indicative of virions to ISVPs conversion.

### 2.2. Antibodies

Mouse monoclonal antibodies 4F2 and 10F6 against reovirus σ3 and μ1 proteins, respectively, were previously described [25]; they were recovered as hybridoma supernatant and used as described in our previous works [26,27]. Primary antibodies against different cellular proteins and secondary peroxidase-conjugated antibodies were obtained from different suppliers and used as recommended, they are listed in Table 1.

### 2.3. Determination of Interferon Induction

Cells were infected at different multiplicity of infection and tissue culture supernatants were collected at different times postinfection as described in the text.

Enzyme-linked immunosorbent assay (ELISA) was then performed on different dilutions of mock-infected or infected cell culture supernatants to determine the concentration of β-interferon (Verikine mouse interferon beta ELISA kit, PBL Assay Science), as previously used [15,28]. Values of optical density were obtained using a microplate reader (BioTEK Elx800); interferon concentration was determined with the standard curve, as recommended by the manufacturer.

### 2.4. Quantification of Cellular and Viral mRNA by RT-qPCR

Cells were infected at different multiplicity of infection and different times as described in the text. Total RNA was extracted with Trizol and further purified using PureLink™ RNA Mini Kit Spin Cartridge, according to the manufacturer’s instructions (Invitrogen, Waltham, MA, USA). Quality and concentration of RNA were verified by optical density on a Thermo Fisher Scientific™ Nanodrop™ One C Spectrophotometer.

Complementary DNA was synthesized from approximately 500 ng of RNA using QuantiNova Reverse Transcription Kit (Qiagen, Hilden, Germany) or QuantiTect Reverse Transcription Kit (Qiagen) according to the manufacturer’s instructions and diluted to 200 ng/μL. Quantitative PCR were performed using a Corbett Research RG-6000 Real Time PCR Thermocycler QIAGEN Rotor-Gene Q. The DNA was amplified using PerfeCTa SYBR^®^ Green FastMix Low ROX from Quantabio. The amplification protocol consisted of an initial activation step of 30 s at 95 °C, followed by 40 cycles of denaturation at 95 °C for 5 s, annealing/elongation at 65 °C for 15 s; data collection was done for 12 s at 72 °C. This was followed by a melt curve step to witness the amplification specificity by increasing the temperature from 72 to 95 °C (1 °C every 5 s).

PCR primer sequences were designed at the Université de Sherbrooke Rnomics Platform using a custom software designed to optimize standard primer design criteria and to certify target specificity. The cellular control genes used are well-known control genes Pum1 and Psmc4 [29], as used in our previous works [30,31], while the viral control genes used are reovirus M1 or reovirus L1, as we have used before [31,32]. Primers for interferon and RIG-I were also previously used [31,32]. All other primers were similarly designed and validated.

All primers were individually resuspended to 100 μM stock solution in ultrapure water and diluted as a primer pair stock solution at 10 μM.

The different primer pairs used are further listed in Table 2.

For each primer pairs, the melting curve was examined. When some of the primer pairs showed a secondary peak, the 17 s annealing/elongation phase was adjusted from 57 °C to 65 °C. A single peak was obtained afterward; this condition was used thereafter.

Each sample was analyzed in triplicate. The threshold was placed to cross the curves where they began their exponential phases. The threshold cycle (Ct) values were obtained and used for the analysis by the delta-delta Ct method formula. The Mock Ct was set as the background and all viruses were compared to it.

### 2.5. Immunoblotting

Proteins were recovered from either infected cells or from infectious virus stocks for analysis by SDS-PAGE and immunoblotting, as previously described [26,27]. Revelation of antigen-antibody complexes was done using peroxidase-conjugated secondary antibody (see Table 1) and chemiluminescent substrate, as recommended by the manufacturer (Pierce SuperSignal West Dura Extended Duration Substrate). Images were obtained and quantitated using a Amersham Imager 600.

### 2.6. Interferon Treatment

L929 cells were treated with different concentrations of commercial mouse β-interferon (PBL Interferon Source), as described in the text. Cells were recovered 15 h later and level of induced proteins were examined by immunoblotting analysis.

### 2.7. Transfection of RNA from Infected Cells

Total RNA was extracted as before and transfected using Lipofectamine MessengerMAX transfection reagent as recommended by the manufacturer (InVitrogen); one microgram of RNA was used for 7.5 × 10^4^ cells seeded in a well of a 12-well plate. In some experiments, oligodT affinity purification was used, as recommended by the supplier (Invitrogen™ Dynabeads™ mRNA Purification Kit Catalog No. 61006), to separate polyadenylated RNA from non-polyadenylated RNA. Polyadenylated RNA was recovered by elution while non-polyadenylated RNA in the flowthrough was left to precipitate overnight at −20 °C after addition of 3 volumes of ethanol, before being resuspended in ultrapure water. Approximately 95% of total RNA was recovered in this fraction. For transfection, the same fraction of either polyadenylated or non-polyadenylated RNA was used, transfected into L929 cells using Lipofectamine MessengerMAX transfection reagent as recommended by the manufacturer (InVitrogen). In each case, one microgram of RNA was used; for polyadenylated RNA, 50 nanograms was used and completed to one microgram with non-polyadenylated RNA from mock-infected cells. Transfected cells were recovered 8 h post-transfection.

### 2.8. Statistical Analysis

Data were compiled in Microsoft Excel (2022) and mean and standard error of the mean were obtained. Statistical significance between the four viruses was established using One-Way ANOVA and Tukey’s multiple comparison tests or Student’s *t*-test using GraphPad Prism Version 9.4.1 for MacOS (2022).

## 3. Results

### 3.1. Further Study of the Impact of μ2 and λ1 on Interferon Induction

It was shown in our previous manuscript that replacing either the μ2 or λ1 protein of T3D^S^ by those of T3D^K^ increases interferon induction in L929 cells while replacing both proteins resulted in a further, more than additive increase. This suggests that the two proteins exert a role in the control of the interferon response, with an apparent synergistic impact.

In the present study, we pursued the study of these different viruses obtained by reverse genetics. The original T3D^S^ virus, derived from our laboratory viral stock, induces low level of interferon and was used as the genetic background with no substitution (indicated as “none” on the different figures). This same genetic background was then used to substitute either the gene encoding μ2, λ1 or both proteins, by those of T3D^K^, the virus derived from the original plasmids used in reverse genetics, that induces higher amount of interferon.

**Figure 1:**
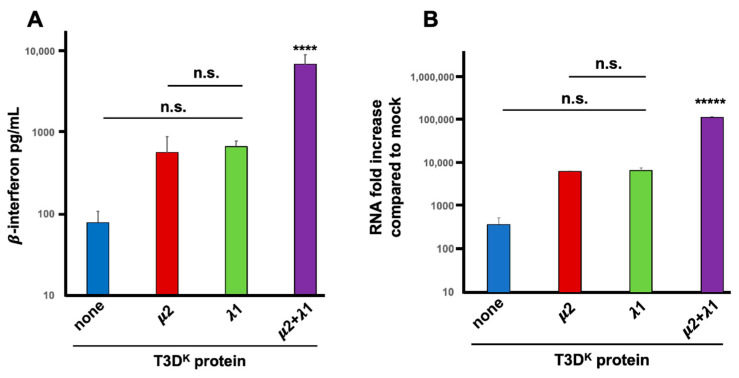
Interferon induction. L929 cells were infected at a multiplicity of infection (MOI) of 20 TCID_50_ units per cell with the different viruses in the T3D^S^ genetic background with either no substitution (none) or substitution of the μ2, λ1, or both proteins of T3D^K^, as indicated, and as described in Materials and Methods. (**A**) Tissue culture supernatants were recovered 15 h post-infection and directly used in a commercial ELISA for β-interferon, as described in Materials and Methods. Results are presented as the mean of 4 independent biological replicates with standard error of the mean. (**B**) Alternatively, total RNA was purified and interferon mRNA was quantified by RT-qPCR. The experiment was repeated twice (biological replicates) and each reaction was performed in triplicates (technical replicates). The data were normalized to the control housekeeping genes, the mock sample was attributed a value of “one” and fold increase compared to mock is shown A mean value was calculated from the technical replicates and the data are presented as the mean value of the two biological replicates with error bars representing the standard error of the mean of the biological replicates. In both panels, statistical significance was established between the calculated means using one-way ANOVA followed by Tukey’s multiple comparison tests. Difference between T3D^S^ and either singly substituted viruses, as well as between singly substituted viruses, was not statistically significant (n.s., *p* > 0.05) using this test. In contrast the doubly substituted virus was significantly different from all the other viruses. (**** *p* < 0.001; and ***** *p* < 0.0005).

Induction experiments in our previous manuscript were performed at a multiplicity of infection of 20 TCID_50_ units per cell, for a single cycle of viral multiplication (15 h). The same results were essentially reproduced herein with multiple replicates. Although slightly different quantitatively, the same conclusion was reached with an approximately 7-fold increase in induction from 70 to roughly 500 pg/mL when either μ2 or λ1 from T3D^K^ was introduced in the T3D^S^ genetic background. A further 11-fold increase was observed when both proteins were substituted, resulting in a more than 80-fold increase compared to T3D^S^ (5700 pg/mL) as shown in Figure 1A. In these experiment, uninfected (mock) cells showed only a very low background signal that was subtracted from the values measured in the supernatants of infected cells. Only when both proteins were substituted was a statistically significant difference observed, with both the T3D^S^ virus and either of the singly substituted viruses. Altogether, these results suggest that although both proteins do affect interferon induction, they exert more than an additive action and that both of them are actually required for maximal impact.

An additional assay was further used to establish if increased levels of interferon is actually due to higher levels of interferon mRNA. Quantitative RT-PCR (RT-qPCR) was performed and values were compared to the small background expression observed in uninfected (mock) cells. This confirmed that either of the singly substituted viruses exhibited a 15 to 20-fold higher induction of interferon mRNA compared to T3D^S^ while the doubly substituted virus had a highly significant 300-fold increase compared to T3D^S^ (Figure 1B). There is thus an increased level of β-interferon mRNA level during infection when either μ2 or λ1 are altered; however, both proteins are clearly acting in synergy during induction of interferon at the mRNA level, either by reducing its level in T3D^S^ or increasing it in T3D^K^.

The same interferon induction experiment was also performed at a low multiplicity of infection (0.1 TCID_50_ unit per cell) for 40 h, in order to measure the induction under conditions of multiple cycle propagation. Again high induction was observed when both T3D^K^ proteins were present. However, under these conditions, induction levels were too low to allow precise measurement of interferon induction with the other viruses, rendering comparisons more hazardous, yet supporting the idea of a synergistic impact of the two proteins.

**Figure 2:**
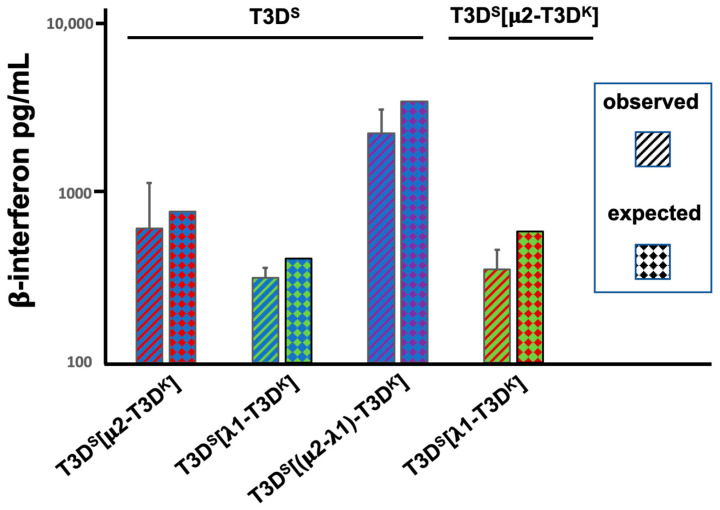
Interferon induction upon co-infection. L929 cells were simultaneously infected with two different viruses at a MOI of 10 TCID_50_ units per cell for each virus, as indicated at the top and bottom of the graph. The tissue culture medium was recovered 15 h post-infection and directly used in the commercial ELISA for β-interferon, as in Figure 1. Results are presented as the average of 4 independent biological replicates with error bars representing the standard error of the mean. The “expected” value presented is the mean of the value for each virus in the pair, as obtained in Figure 1A.

We next investigated whether the μ2- and λ1-associated induction (or lack of) phenotypes could be dominant or recessive. Cells were simultaneously co-infected at a MOI of 10 for both the T3D^S^ virus and the different viruses harboring either the μ2, λ1 or both proteins from T3D^K^ in the T3D^S^ background. A similar experiment was done by co-infecting with the two singly substituted viruses. The induction of interferon measured by ELISA was then compared with the expected value, as determined by the data of Figure 1, if induction was simply intermediate between the two co-infecting viruses. This was actually the case in all pairs of viruses. Altogether, once again, the results strongly suggest that both proteins are independently involved in the control of interferon induction and that the presence of both protein is necessary for the full phenotype (Figure 2). The simplest explanation is that each co-infecting virus independently induce interferon, at a level relying on the nature of its μ2- and λ1 proteins. Interestingly, a similar observation of distinct behavior of incoming virions during co-infection was also reported by another group, although the assays used and genetic backgrounds of the viruses under study were different, making direct comparisons difficult [33].

### 3.2. The Impact of μ2 and λ1 on Levels of Proteins of the Interferon Signaling Network

**Figure 3:**
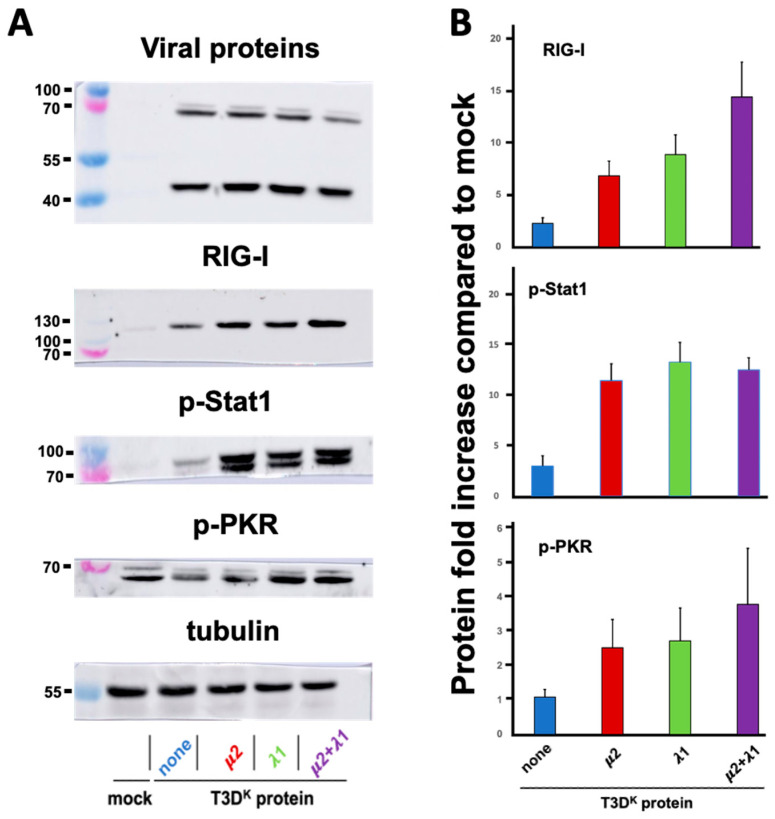
Increased levels of various proteins along the interferon signaling network. L929 cells were either mock-infected or infected at a MOI of 10 TCID_50_ units per cell with the T3D^S^ virus or the T3D^S^ virus harboring the μ2, λ1 or both μ2 and λ1 of T3D^K^ in the T3D^S^ background, as indicated; again, none refers to the T3D^S^ virus with no T3D^K^ proteins substituted. Cells were incubated for 15 h before being recovered and the different proteins analyzed by immunoblotting, as described in Materials and Methods, using antibodies listed in Table 1. (**A**) Representative immunoblots of two or three independent biological replicates are presented. In each case, viral proteins were also immunodetected using a combination of monoclonal antibodies against viral proteins σ3 at a predicted molecular weight of 41 kDa and μ1, detecting the μ1C protein cleavage at a predicted molecular weight of 72 kDa. A control for loading using tubulin was also used. (**B**) Quantitation of the replicates (three or four) is presented. For each replicate, the value of mock-infected cells was fixed as “one” and all samples were adjusted accordingly, taking into account small differences in the values of tubulin. Molecular weight standards in kilodaltons are indicated on the left of each panel. All data are presented as the means with error bars representing the standard error of the mean.

In an effort to better understand the mechanism underneath the independent and apparently synergistic effect of μ2 and λ1 on differential interferon induction, we chose to first examine proteins along the interferon signaling network; this included some phosphoproteins for which transcriptional analysis is not adequate. We chose to examine the RIG-I sensor (or pattern-recognition receptor, PRR), phospho-Stat1 involved in signaling from the interferon receptor and phospho-PKR, as a classical protein product from an interferon-stimulated gene [34,35]. Cells were infected with either one of the different viruses, proteins were recovered and analyzed by immunoblotting (Figure 3).

Increase in RIG-I upon T3D^S^ infection could be seen as it was augmented by approximately fourfold compared to T3D^S^ when either μ2 or λ1 from T3D^K^ was substituted. However, as previously observed for interferon induction, combination of both μ2 and λ1 results in a more than additive effect on RIG-I level, with a more than 15-fold increase compared to T3D^S^. An increased level of phospho-Stat1 was also observed with a more than fourfold increase with either the singly substituted or doubly substituted viruses with no apparent differences between these viruses. Finally, for phospho-PKR, we could observe a limited similar induction with either T3D^S^ or the different viruses, even compared to mock-infected cells. It thus seems that only RIG-I presents a pattern of increase that parallels that of interferon.

### 3.3. Kinetic of Increase in RIG-I and Phospho-Stat1 during Viral Propagation

**Figure 4:**
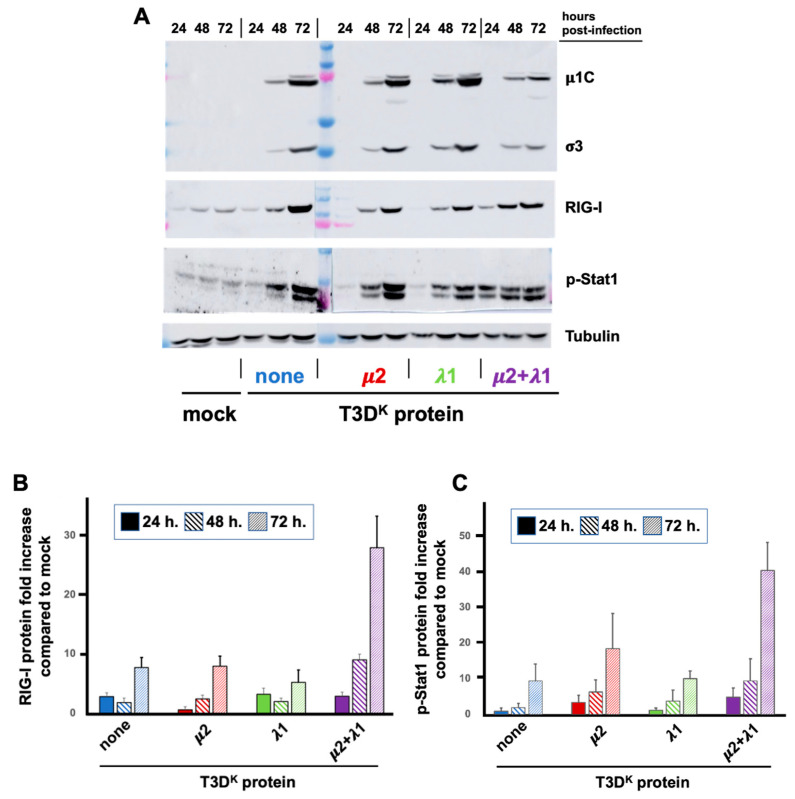
L929 cells were infected at a MOI of 0.05 TCID_50_ units/mL with the different viruses in the T3D^S^ genetic background with either no substitution (none) or substitution of the μ2, λ1, or both proteins of T3D^K^, as indicated and as described in Materials and Methods. Proteins were recovered from infected cells at different times post-infection (24, 48 and 72 h). (**A**) Immunoblotting analysis was performed against either virus proteins (μ1C and σ3), RIG-I, phospho Stat1 or tubulin, as indicated. The results of a representative experiment is presented. (**B**,**C**) Immunoblots of three biological replicates were quantified for RIG-I in panel b and phospho-Stat1 in panel c; small correction from different loading, using tubulin was first used and then the increase in RIG-I or phospho-Stat1 was analyzed, taking into account the different amounts of viral proteins. The values obtained from T3D^S^ with no protein from T3D^K^ substituted were considered as “one” for all three different times.

In the previous experiment, there was no apparent effect on the synthesis of viral proteins in the presence of increased levels of RIG-I and phospho-Stat1. However, these experiments were performed for a single viral multiplication cycle in conditions where viral propagation is minimal. We thus further examined the synthesis of viral proteins in parallel with RIG-I and phospho-Stat1 over time at a lower multiplicity of infection (0.05 TCID_50_ units/cell), thus allowing viral propagation (Figure 4). The differential induction of RIG-I between viruses could not be observed at 24 h under these conditions although some, limited, increase was observed with all viruses at this time point. At 48 h and 72 h, there was a highly significant difference between the doubly substituted virus and either of the singly substituted viruses or T3D^S^ when one takes into account the relative level of viral proteins. It appears that viral propagation is limited by induction of the interferon response only in the case of the doubly substituted virus. Once again, although an effect is observed on RIG-I with both singly substituted virus, it was quite limited compared to the doubly substituted virus and with no significant impact on viral propagation.

In the case of phospho-Stat1, the increase could barely be seen at 24 h except for the doubly substituted virus. Levels continue to increase at 48 and 72 h for the different viruses, close to maximal level is already reached at 24 h for the doubly substituted virus. However, when taken into account the levels of viral proteins, the phospho-Stat1 level is both higher at early times in the doubly substituted virus and continues to increase with time reflecting inhibition of its replication. However, the difference between the doubly substituted virus and both T3D^S^ and the singly substituted viruses is not as important (and does not reach statistical significance) compared to the impact on RIG-I. This suggests that it is rather the consequence of interferon secretion at a saturating level rather than a direct consequence of the presence or absence of two T3D^K^ viral proteins.

### 3.4. Effect of Interferon on RIG-I and Phospho-Stat1 in the Absence of Viral Infection

Considering previous results, we next examined if the higher level of RIG-I and phospho-Stat1 proteins in singly or doubly substituted viruses, concomitant with increased in β-interferon protein and RNA, could be solely and indirectly due to these increased interferon levels. Commercially available β-interferon was thus added to the cells at either the concentration approximately found in the medium of T3D^S^-infected cells (1 IU/mL or 50 pg/mL), or in the approximate concentration found in the medium of cells infected with either the singly substituted viruses (10 IU/mL or 500 pg/mL) or the doubly substituted virus (100 IU/mL or 500 pg/mL). Cells were recovered 15 h later and analyzed by immunoblotting against RIG-I and phospho-Stat1.

**Figure 5:**
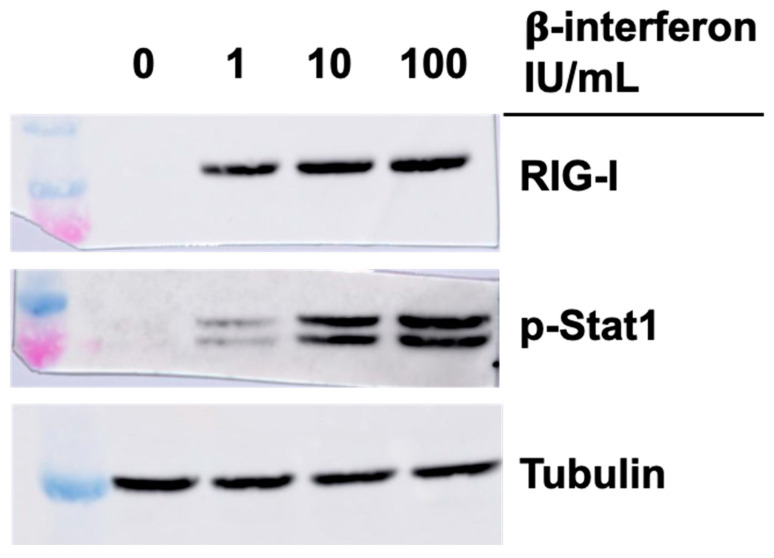
Impact of interferon treatment on RIG-I and phospho-Stat1 in L929 cells. Cells were either mock-treated or treated with varying concentration of commercial β-interferon. Proteins were analyzed by immunoblotting 15 h after treatment, as indicated.

As shown in Figure 5, interferon treatment did increase RIG-I level but this rapidly saturates at low concentration and it is thus unlikely that the presence of interferon is responsible solely for the observed increase during infection. In the case of phospho-Stat1, the increase is similar to what is observed with the different viruses with higher levels at an interferon concentration corresponding to the singly substituted viruses, with no real difference when interferon concentration was further increased. It is thus likely that observed increase during infection is due for the most part to increased interferon secretion by the infected cells.

### 3.5. The Impact of μ2 and μ1 on Transcription of Genes of the Interferon Signaling Network

We then further examined the impact of infection on transcription of various cellular genes known to be involved in the host-cell response to viral infection. Numerous works in the literature has examined the impact of reovirus on the transcriptome using different approaches [36,37,38,39,40]; however, the cell lines used and the viral isolate used is variable making comparisons somewhat difficult.

In previous work, we examined the impact of T3D^S^ on L929 cells transcriptome using RNAseq [32]. Among the more than 250 transcripts that were induced more than tenfold under the experimental conditions used, many of these were well-known components of the interferon signaling or response network. For example, RIG-I (also known as DDX58), IRF-7, Stat1, OasL2 and Ifit1 were all induced between roughly 20-fold (Stat1) and more than 900-fold (Ifit1) despite the fact that T3D^S^ does not strongly induce interferon.

**Figure 6:**
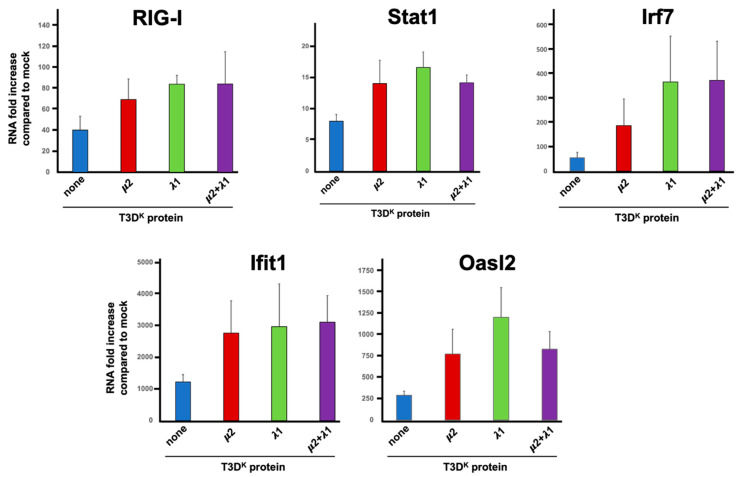
Induction of mRNA of genes of the interferon signaling network. L929 cells were infected at a MOI of 10 TCID_50_ units/cell with the different viruses in the T3D^S^ genetic background with either no substitution (none) or substitution of the μ2, λ1, or both proteins of T3D^K^, as indicated and as described in Materials and Methods. Total RNA was purified 15 h post-infection. The mRNA for the different indicated genes was quantitated by RT-qPCR. The experiment was done twice (biological replicates) except for RIG-I and Oasl2 done in triplicates, each reaction was then performed in triplicates (technical replicates). The data were normalized to the control housekeeping gene, the mock sample was attributed a value of “one”. A mean value was calculated from the technical replicates and the data are presented as the mean value of the two biological replicates. All error bars represent the standard error of the mean.

Based on these data, the induction level of given mRNAs was thus compared by RT-qPCR upon infection with either T3D^S^ or the viruses, harboring either μ2, λ1 or μ2 and λ1 combination of T3D^K^ in the T3D^S^ background. Somewhat surprisingly, although RIG-I mRNA level was increased at least 30-fold upon infection as expected, there was only a small twofold increase with either of the singly or doubly substituted viruses compared to T3D^S^ (Figure 6); it thus appears that the increase seen at the protein level is not a direct consequence of an increase at the transcriptional level.

The induction of Stat1 upon T3D^S^ infection was also observed at the transcriptional level although it was relatively modest; it was more evident at the protein level for phospho-Stat1, suggesting that phospho-Stat induction with T3D^S^ is limited both at the level of total induction and phosphorylation level. As for the protein induction, either the singly and doubly substituted viruses have an increased level of induction, although only approximately twofold. This appears quite low compared to the increased phospho-Stat level, suggesting that both expression and phosphorylation of this protein are affected. As for RIG-I, there was no synergistic, or even additive, effect between μ2 and λ1.

The major transcriptional interferon regulatory factor 7 (Irf7) was also examined and shown to be induced approximately 50-fold by infection; there was a statistically significant 3.5 and 6.5 increased induction with the single reassortant for μ2 and λ1, respectively, but no additive effect between the two proteins. The difference between μ2 and λ1 was not statistically significant but nevertheless suggest that the two proteins may exert a slightly different effect on the interferon signaling network with λ1 being the major determinant.

Two different interferon-stimulated genes, Ifit1 and Oasl2, were next examined and shown to be more strongly induced, 300-fold and 1200-fold, respectively, upon infection, as expected from previous RNAseq data. Induction was further increased but only by two to fourfold upon substitution of either μ2 or λ1 with no apparent further increase in the doubly substituted virus. This suggests that the level of interferon induction by T3D^S^ although relatively limited is sufficient to reach near-saturating effect on induction of interferon-stimulated genes acting as effectors of the interferon response. This is also consistent with our previous observation [31] that addition of 1 international unit of β-interferon per mL is sufficient to reach near maximal levels of DDX60 and Mx1 as representative interferon-stimulated genes.

Altogether, the results suggest that while the initial induction of interferon is affected by μ2 and λ1, the resulting impact on the interferon signaling network is actually limited, at least under these conditions, consistent with the limited impact on viral protein synthesis in one replication cycle.

### 3.6. Differential Effect of Partially Uncoated Virions, ISVPs, Compared to Virions

It has been previously hypothesized that genomic RNA, although generally considered as retained in the viral particles during the whole viral multiplication cycle, could in fact be partly exposed by leaking during viral entry [41]. This suggests that complete virions and partially uncoated ones, ISVPs, could exert a different effect on interferon induction due to differences in intracellular entry pathways, ISVPs largely bypassing endosomes [35,42]. More recent data confirm this idea showing that ISVPs are significant less potent inducers than virions, possibly due to differences in recognition by endosomal and cytoplasmic sensors and differences in exposure of viral genomic dsRNA [43,44,45,46]. We thus examined if the difference in interferon induction between T3D^S^ and the virus harboring both μ2 and λ1 of T3D^K^ is retained or abolished upon infection by ISVPs derived from these two viruses.

**Figure 7:**
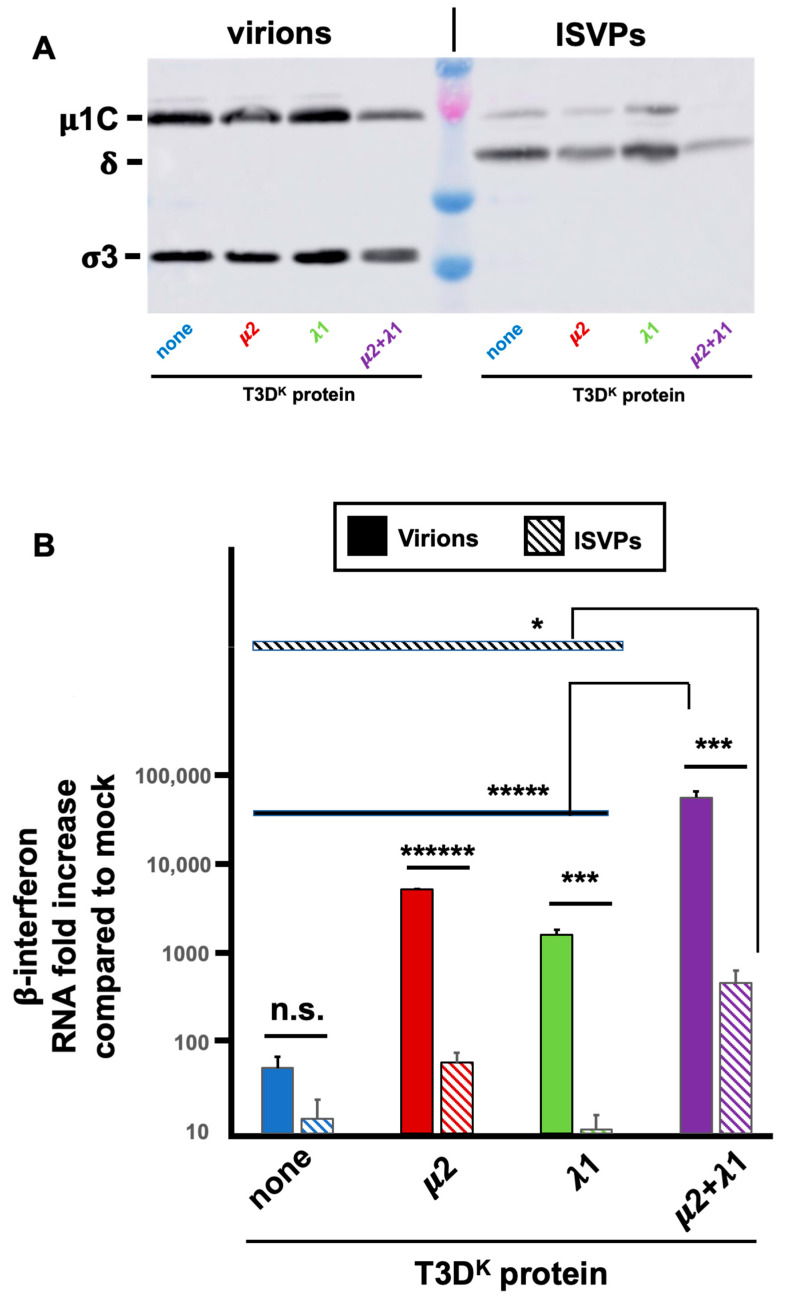
Comparison of virions and ISVPs. Infectious virus stocks were prepared and treated with chymotrypsin as described in Materials and Methods and used to infect L929 cells at a MOI of 10 TCID_50_ units/cell. The different viruses in the T3D^S^ genetic background with either no substitution (none) or substitution of the μ2, λ1 or both proteins of T3DK were compared. Total RNA and proteins were recovered and analyzed, as before and as described in Materials and Methods. (**A**) Aliquots of the virions and ISVPs stocks were examined by immunoblotting showing the disappearance of σ3 and μ1C to δ conversion in ISVPs for all 4 different viruses. (**B**) RT-qPCR was performed to detect β-interferon and viral M1 RNA. The experiment was done three times (biological replicates) for ISVPs and twice for virions; each reaction was then performed in triplicates (technical replicates). The data were normalized to the control housekeeping gene, the mock sample was attributed a value of “one”. A mean value was then calculated from the technical replicates and the data are presented as the mean value of the biological replicates, taking into account small variations in viral mRNA. The induction by the T3D^S^ virions being initially considered as “one”. Error bars represent the standard error of the mean. Statistical significance between virions and ISVPs was established by Student’s *t*-test. Statistical significance between the doubly substituted virus and all the other viruses, the different viruses, either as virions or ISVPs, was established using ANOVA followed by Tukey’s multiple comparison tests. ****** *p* < 0.0001, ***** *p* < 0.0005, *** *p* < 0.005, * *p* < 0.05, n.s.: *p* > 0.05.

ISVPs were prepared from infectious virus stocks, as described in Materials and Methods and compared with mock-treated samples from the same virus stocks as complete virions. Virions to ISVPs conversion was confirmed, as revealed by disappearance of σ3 and near complete μ1C to δ cleavage; most of the virions appear to be converted to ISVPs although a residual signal of μ1C suggests that there is a small amount of incompletely converted virions remaining (Figure 7A). Virions and ISVPs were frozen and titers of aliquots were determined to ensure that infection will be performed at similar multiplicity of infection for both virions and ISVPs from the different viruses.

Infection was then performed with either virions or ISVPs and RNA harvested to be subjected to RT-qPCR for β-interferon mRNA as well as viral mRNA. Consistent with previous reports, there was a strongly reduced induction of β-interferon mRNA by ISVPs compared to virions from the same virus stocks (Figure 7B). Interestingly, as virions, there was a 50 to a hundredfold increase in interferon induction by the singly substituted viruses and more than a thousandfold by the doubly substituted virus. As ISVPS, the difference is essentially abolished for singly substituted virus and reduced to at most 30-fold for the doubly substituted virus. Furthermore, the small remaining difference can probably be attributed to remaining virions in the ISVPs. It thus appears that the difference of interferon induction between the different viruses harboring either μ2 or λ1 of T3D^K^ is mostly lost when infection is performed by ISVPs.

**Figure 8:**
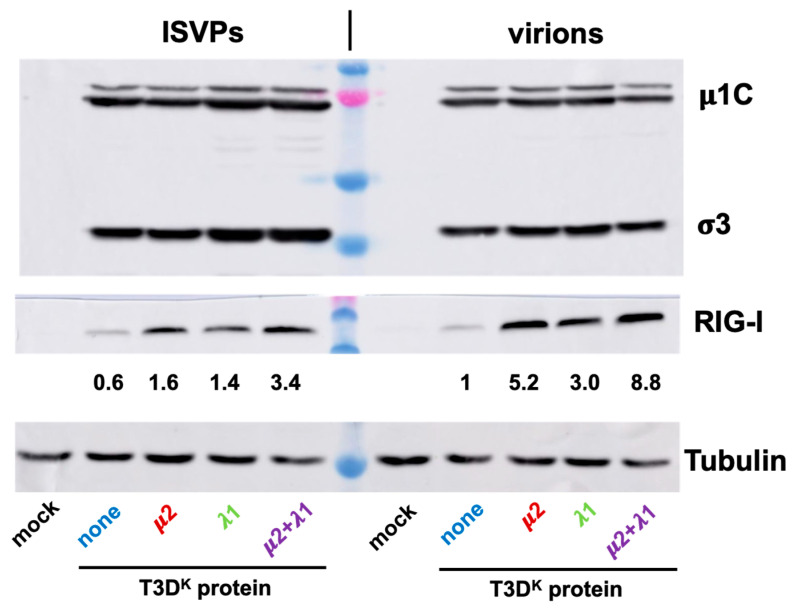
Comparison of virions and ISVPs. Infectious virus stocks were prepared and treated with chymotrypsin as in Figure 7, and used to infect L929 cells. Proteins were recovered and analyzed for viral proteins, RIG-I and tubulin, as indicated. Relative RIG-I values were calculated taking into account small differences in tubulin and levels of viral proteins, the value for T3D^S^ virions being fixed as “one”.

Levels of RIG-I upon infection with either virions or ISVPs was also briefly examined. In preliminary experiments, we noticed that infection by ISVPs, as estimated by levels of viral proteins, was more efficient than that of virions. We thus adjusted multiplicity of infection to reach similar levels of viral proteins with the different ISVPs and virions (Figure 8, upper panel). As somewhat expected, RIG-I levels were higher upon infections by virions for all 4 viruses and much lower with T3D^S^ than with the other viruses (Figure 8, middle panel). As for interferon induction, the difference between the different viruses was reduced but not completely abolished when ISVPs were used to infect. Once again, it thus appears that RIG-I level parallels that of interferon induction.

### 3.7. Induction of Interferon by RNA from Infected Cells

Since μ2 and λ1 are inner capsid proteins probably, or putatively, involved in either viral mRNA synthesis or capping [47,48] the ability of viral RNA present in infected cells to stimulate induction of interferon was next examined. To do so, total RNA was extracted and first separated in polyadenylated and non-polyadenylated RNA. Since reovirus RNA is devoid of the poly(A) tail, this will allow to largely deplete the non-polyadenylated fraction from cellular mRNAs that harbor such a 3′-end tail. This was considered necessary at first to ensure that any difference observed between the RNA from cells infected by the different viruses will not be due to differences in induced cellular mRNA present in the cells, but rather to differences in viral RNA.

The fractions were verified for their content of viral RNA and cellular mRNA. As expected, there was a strong, more than 8000-fold depletion of cellular gene mRNA used as a control (Psmc4) in the non-polyadenylated fraction while a large proportion of the viral mRNA is retained in this fraction. As expected, transfection of RNA from mock-infected cells did not induce β-interferon mRNA; in contrast, when RNA was extracted from T3D^S^-infected cells, the non-polyadenylated fraction containing viral RNA resulted in a strong induction with no effect of the polyadenylated RNA fraction used at the same ratio; this convincingly ruled out the possibility that induced cellular mRNA could be involved in the observed effects. Thereafter, total RNA or non-polyadenylated RNA was thus indifferently used in these experiments.

**Figure 9:**
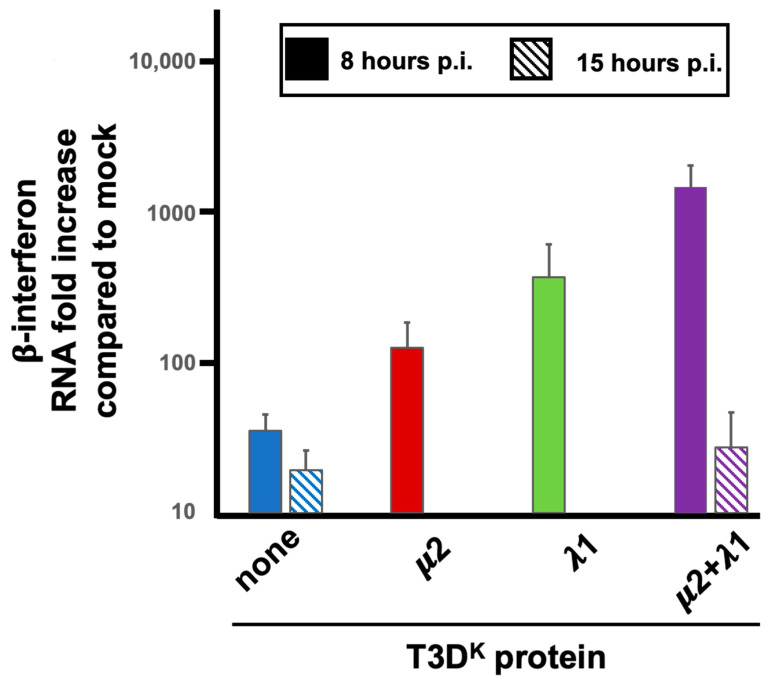
Interferon mRNA induction by RNA recovered from infected cells. L929 cells were infected at a MOI of 10 TCID_50_ units/cell with the different viruses in the T3D^S^ genetic background with either no substitution (none) or substitution of the μ2, λ1, or both proteins of T3D^K^, as before. Total RNA was recovered at either 8 h or 15 h post-infection with the different viruses, as indicated and as described in the text. RNA was introduced by transfection, as described in Materials and Methods, and cells were recovered 8 h post-transfection. The experiment was repeated twice for singly substituted viruses (biological replicates) with RNA extracted at 8 h post-infection and each quantification reaction was performed in triplicates (technical replicates). The experiment with both other viruses was done in biological triplicates with RNA recovered at early or late time postinfection. The data were normalized to the control housekeeping gene, the mock sample was attributed a value of “one”. Even though clear difference was observed between RNA recovered at 8 h for the different viruses, statistical significance was not reached in these experiments (*p* = 0.07).

We then first concentrated our efforts by comparing T3D^S^ and the doubly substituted virus harboring both μ2 and λ1 from T3D^K^, comparing the ability of the RNA extracted at early (8 h) and late (15 h) postinfection. There was no apparent difference between the two viruses when the RNA used was obtained from late-infected cells (Figure 9). The difference in induction of interferon upon infection with these low- and high-inducing viruses thus appear to be independent of the amount or nature of viral RNA present in the infected cells at late-time post infection. In contrast, when induction by RNA extracted at early times was examined, induction was increased by more than 50-fold when RNA was extracted from cells infected by the doubly substituted virus rather than T3D^S.^ (Figure 9). The induction by RNA from T3D^S^-infected cells was essentially identical when RNA was recovered either at 8 or 15 h, when considering the much lower level of viral RNA present at 8 h, as measured by RT-qPCR.

We then verified if RNA extracted from cells infected by the two singly substituted viruses behave as intermediate between T3D^S^ and the doubly substituted virus. It turns out to be the case (Figure 9), indicating that the nature of the RNA inducer is, once again, dependent on both μ2 and λ1.

## 4. Discussion

In the present study, we revisited and extended our previous results showing that both μ2 and λ1 are involved in the control of interferon synthesis. We further showed control of induction of β-interferon mRNA at the transcriptional level, and that the two proteins exert a combined action resulting in maximal impact on interferon induction. The intermediate level of induction observed upon co-infection with high- and low- interferon-inducing virus suggests that the two proteins do not exert their effect by their presence in the infected cell, but rather by their presence in the infecting viral particles.

Analysis of sequences available for both the μ2 and λ1 proteins indicates that for the three polymorphisms examined herein (position 208 and 342 of μ2 and position 500 in λ1), the sequence of T3D^S^ is by far the most frequent and found in more than 90% of the viruses. This suggests that the low induction phenotype of T3D^S^ is in fact the natural wild-type phenotype and that higher inducing variants were probably selected upon virus passage in tissue culture. In fact, it was noticed that L929 cells grown in suspension culture exhibit constitutively active PKR that makes them inappropriate to discriminate between reoviruses variants exhibiting different levels of sensitivity to the interferon response [49]; this could partly explain the selection of viruses with increased induction of—or sensitivity to—interferon as T3D^K^ [15].

**Figure 10:**
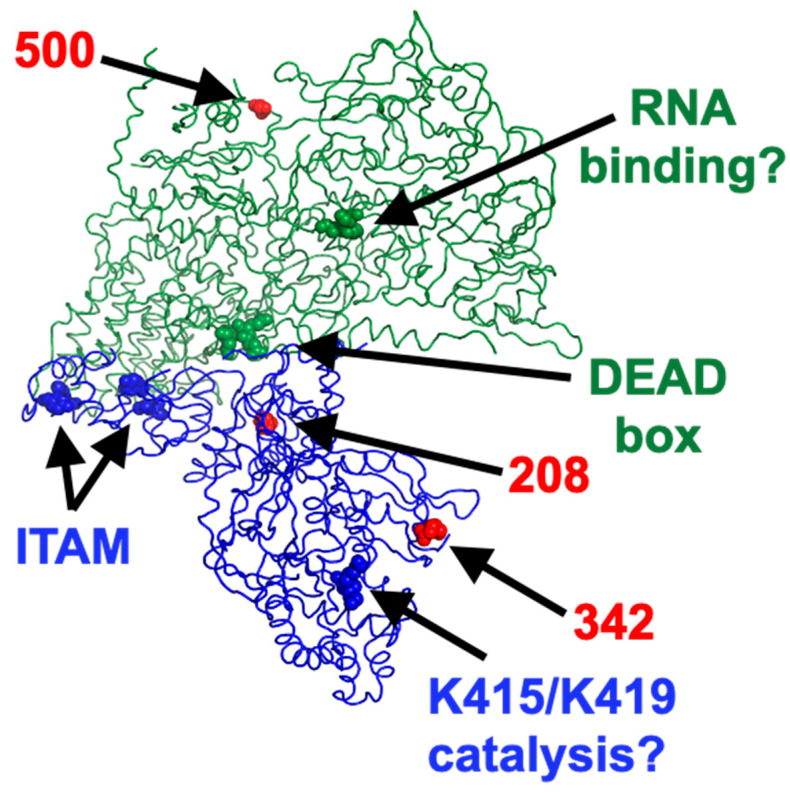
The structure was obtained from ncbi under the accession number 7elh. For simplicity a single molecule of both μ2, in blue, and λ1, in green is presented as ribbon structure and viewed from the core’s interior. Residues of interest are represented by spheres, as indicated and described in the text. Red spheres are the three residues of μ2 and λ1 that differ between T3D^S^ and T3D^K^. The figure was prepared using PyMOL© version 2.5.1.

Recent data have allowed to obtain detailed structure analysis of the μ2 protein in the context of the viral particle [50] thus allowing visualization of the position of the amino acid substitutions on both μ2 and λ1 (Figure 10). Interestingly, the DEAD box of λ1, the previously described ITAM of μ2, and the 208 polymorphic position on μ2 are all located close to the interface between the two proteins. In contrast, the 342 polymorphic position of μ2 is located close to K415/K419 amino acids involved in the μ2 NTPase activity, at least in vitro. It will be interesting to examine which of the polymorphism at position 208, already known to be associated with multiple phenotypes [30,51,52], or the 342 position, for which there is to our knowledge no associated phenotype, is actually associated with differences in interferon induction. It should be noted that the substitution of proline for a serine at the 208 position was shown to result in a protein exhibiting a thermosensitive phenotype for some of its function [53]; it may simply be the case in its role for the control of the interferon induction.

Both μ2 and λ1 have been proposed to be responsible for the first step of viral mRNA capping [54,55], namely the removal of the 5′ terminal γ-phosphate by an RNA triphosphatase activity (recently reviewed in: [47,48]). Both proteins by themselves can exert such an activity in vitro and exhibit similar K_m_ for removal of the γ-phosphate from 5′-triphosphorylated RNA. This raises the possibility that either μ2 and λ1 substitution may have a similar impact on the viral transcription complex. It cannot be excluded that the function may somehow redundant or complementary in the viral core.

Although it is not expected that λ1 could have an impact on viral disassembly from virions to infectious subviral particles then to transcriptionally active core, recent data have shown that λ1 contributes to inner capsid stability [56]. Therefore, it may be of importance in the release of a certain amount of dsRNA resulting in recognition by the host cell antiviral response. Interestingly, the single amino acid difference in λ1 between T3D^S^ and T3D^K^ is located well away of μ2 but rather at the external surface, suggesting a possible impact on the core structure (Figure 10).

While λ1 is only involved in induction of the interferon response, rather than sensitivity of the virus to this response, the μ2 protein does have an impact on both induction and sensitivity [15]. This complicates the analysis to a certain extent by introducing a bias if viral multiplication is affected by the interferon response. This is likely part of the explanation for the decreased viral proteins level for the doubly substituted virus under conditions allowing viral propagation, although this effect was not observed with the μ2 singly substituted virus. It will be of interest to attempt to separate the impact of μ2 on induction and sensitivity to interferon, but this might not be possible to achieve. The μ2 protein is not believed to have a role in early events during infection. However, it seems to be of importance in late events leading to different efficiency in mature, genome-containing, particle assembly, at least in some cell types [57]. Interestingly, it has also been reported that the presence of the cap structure does affect RNA packaging [58]; suggesting that varying efficiency of cap synthesis, due to polymorphisms in different viral proteins involved, could affect the efficiency of packaging or the nature of the 5′-terminal end of packaged RNA.

Altogether, the data presented herein thus support a model in which both μ2 and λ1 individually control interferon induction and that their combined synergistic effect is required for maximal impact during early events. The distinct and separate behavior of the virions, combined with the data on ISVPs and induction by early RNA support this idea. Two different explanations come to mind. Either the two proteins affect viral disassembly and exposure of viral RNA or, alternatively, the nature of the viral RNA in the incoming particles is different due to different synthesis or packaging at the previous cycle. In both cases the proximity of both proteins and their similar enzymatic activity are consistent with a possible redundant or complementary role.

Interestingly, in addition to our work, there are different evidence suggesting that μ2, λ1 and even λ2 could contribute to common phenotypes and/or functionally interact [33,53,59], although mechanisms involved remain somewhat elusive. In the present study, we demonstrated that transfection of RNA from infected cells is a promising approach to study determinants of interferon induction. In the future, it could be of interest to determine the 5′-terminal end of viral mRNA and genomic RNA at early and late time points and the kinetics of the production of the RNA induced of the interferon network. A more detailed kinetics of viral disassembly for the viruses studied herein could also further contribute to our understanding of viral determinants involved. In addition, combining the amino acid substitutions of μ2 and λ1 with those of either λ2 or σ1s known to affect interferon sensitivity without affecting its induction [14,15,28], should certainly be envisaged to better comprehend viral determinants and mechanisms involved in these two critical steps of the innate immune response.

## Figures and Tables

**Table 1:** Antibodies used in this study.

Antibody	Supplier (Catalog #)	Dilution Used
Rig-I (D14G6) Rabbit mAb	Cell Signaling Technology (#3743)	1:1000
Phospho-Stat1 (Tyr701) (ST1P-11A5) Mouse mAb	Fisher Scientific/Invitrogen (REF: 33-3400)	1:1000
Phospho-PKR (Thr451) Rabbit polyclonal Ab	Fisher Scientific/Invitrogen (REF: 44-668G)	1:1000
Anti-Tubulin antibodies from Rabbit	ICN Biomedicals	1:2000
Donkey anti-rabbit IgG, ECL HRP-linked whole antibody	GE Healthcare (NA934V)	1:2000
Goat anti-mouse IgG, (H + L) HRP-linked Antibody	Thermo Scientific™ Pierce™	1:2000

**Table 2:** Primers used in this study.

Gene Name	Primer Name	Sequence (5′–3′)
Pum1 ^1^	Psmc4.mm.qref.F1	CCCAGGAGGAGGTGAAGCGG
	Psmc4.mm.qref.R1	GGTCGATGGTACTCAGGATGCG
Psmc4 ^1^	Pum1.mm.qref.F	TGCCAGTCTCTTCCAGCAGCA
	Pum1.mm.qref.R1	TGATTTGGGGTCAAAGGACGTTGG
M1 ^2^	ReoM1-1F	AGCGCGCAGCCTAAATGGTT
	ReoM1-1R	ACGTAGATGCCGGGTCTGCT
L1 ^2^	L1-1F	GGCAAAGACGGTGTCGGGTC
	L1-1R	TGCGTCCGCTTCTGACTCCT
Ifnb	Ifnb1.qmm.F3	ACACTGCCTTTGCCATCCAAGA
	Ifnb1.qmm.R3	ACACTGTCTGCTGGTGGAGTTCA
Ifit1	Ifit1.qmm.F1	ACCCAGAGAACAGCTACCACCT
	Ifit1.qmm.R1	CAGCTTCCATGTGAAGTGACATCTCA
Oasl2	Oasl2.qmm.F1	GCTGACCCCACCAACAACCTG
	Oasl2.qmm.R1	TCTCACCTGAACATCCCTCGCT
Stat1	Stat1.qmm.F1	GCTGTACGATGACAGTTTCCCCA
	Stat1.qmm.R1	CGGATGGTCGCAAACGAGACAT
Ddx58 (RIG-I)	Ddx58.qmm.F1	AACTTGCTTTGGAGAAAGACAACAG
	Ddx58.qmm.R2	TCTGACACTCTGGCTCTTCCTGA
Irf7	Irf7.qmm.F1	GACTTCAGCACTTTCTTCCGAGAA
	Irf7.qmm.R2	ACAAGTCTTGCCCAAAACCCAG

^1^ Housekeeping genes, ^2^ Viral control genes, F for forward primers and R for reverse primers.

## Data Availability

Not applicable.

## References

[1] Dermody T.S., Parker J.S.L., Sherry B.L. (2013). Field’s Virology.

[2] Fields B.N. (1972). Genetic manipulation of reovirus--a model for modification of disease. N. Engl. J. Med..

[3] Fields B.N. (1992). Studies of reovirus pathogenesis reveal potential sites for antiviral intervention. Adv. Exp. Med. Biol..

[4] Sharpe A.H., Fields B.N. (1985). Pathogenesis of viral infections. Basic concepts derived from the reovirus model. N. Engl. J. Med..

[5] Chakrabarty R., Tran H., Selvaggi G., Hagerman A., Thompson B., Coffey M. (2015). The oncolytic virus, pelareorep, as a novel anticancer agent: A review. Investig. N. Drugs.

[6] Clements D., Helson E., Gujar S.A., Lee P.W. (2014). Reovirus in cancer therapy: An evidence-based review. Oncolytic Virother..

[7] Zhao X., Chester C., Rajasekaran N., He Z., Kohrt H.E. (2016). Strategic combinations: The future of oncolytic virotherapy with reovirus. Mol. Cancer Ther..

[8] Geoffroy K., Bourgeois-Daigneault M.C. (2020). The pros and cons of interferons for oncolytic virotherapy. Cytokine Growth Factor Rev..

[9] Katsoulidis E., Kaur S., Platanias L.C. (2010). Deregulation of interferon signaling in malignant cells. Pharmaceuticals.

[10] Matveeva O.V., Chumakov P.M. (2018). Defects in interferon pathways as potential biomarkers of sensitivity to oncolytic viruses. Rev. Med. Virol..

[11] Stojdl D.F., Lichty B.D., Paterson J.M., Power A.T., Knowles S., Marius R., Reynard J., Poliquin L., Atkins H., Brown E.G. (2003). VSV strains with defects in their ability to shutdown innate immunity are potent systemic anti-cancer agents. Cancer Cell.

[12] Li Q., Tan F., Wang Y., Liu X., Kong X., Meng J., Yang L., Cen S. (2022). The gamble between oncolytic virus therapy and IFN. Front. Immunol..

[13] Rudd P., Lemay G. (2005). Correlation between interferon sensitivity of reovirus isolates and ability to discriminate between normal and Ras-transformed cells. J. Gen. Virol..

[14] Sandekian V., Lemay G. (2015). A single amino acid substitution in the mRNA capping enzyme λ2 of a mammalian orthoreovirus mutant increases interferon sensitivity. Virology.

[15] Lanoie D., Lemay G. (2018). Multiple proteins differing between laboratory stocks of mammalian orthoreoviruses affect both virus sensitivity to interferon and induction of interferon production during infection. Virus Res..

[16] Kemp V., Hoeben R.C., van den Wollenberg D.J. (2015). Exploring reovirus plasticity for improving its use as oncolytic virus. Viruses.

[17] Mohamed A., Johnston R.N., Shmulevitz M. (2015). Potential for improving potency and specificity of reovirus oncolysis with next-generation reovirus variants. Viruses.

[18] Steyer A., Gutiérrez-Aguire I., Kolenc M., Koren S., Kutnjak D., Pokorn M., Poljšak-Prijatelj M., Rački N., Ravnikar M., Sagadin M. (2013). High similarity of novel orthoreovirus detected in a child hospitalized with acute gastroenteritis to mammalian orthoreoviruses found in bats in Europe. J. Clin. Microbiol..

[19] Uehara A., Tan C.W., Mani S., Chua K.B., Leo Y.S., Anderson D.E., Wang L.-F. (2018). Serological evidence of human infection by bat orthoreovirus in Singapore. J. Med. Virol..

[20] Yamamoto S.P., Motooka D., Egawa K., Kaida A., Hirai Y., Kubo H., Motomura K., Nakamura S., Iritani N. (2020). Novel human reovirus isolated from children and its long-term circulation with reassortments. Sci. Rep..

[21] Yang X.-L., Tan B., Wang B., Li W., Wang N., Luo C.-M., Wang M.-N., Zhang W., Li B., Peng C. (2015). Isolation and identification of bat viruses closely related to human, porcine and mink orthoreoviruses. J. Gen. Virol..

[22] Kobayashi T., Antar A.A., Boehme K.W., Danthi P., Eby E.A., Guglielmi K.M., Holm G.H., Johnson E.M., Maginnis M.S., Naik S. (2007). A plasmid-based reverse genetics system for animal double-stranded rna viruses. Cell Host Microbe.

[23] Danis C., Lemay G. (1993). Protein synthesis in different cell lines infected with orthoreovirus serotype 3: Inhibition of host-cell protein synthesis correlates with accelerated viral multiplication and cell killing. Biochem. Cell Biol..

[24] Bergeron J., Mabrouk T., Garzon S., Lemay G. (1998). Characterization of the thermosensitive ts453 reovirus mutant: Increased dsRNA binding of sigma 3 protein correlates with interferon resistance. Virology.

[25] Virgin H.W.t., Mann M.A., Fields B.N., Tyler K.L. (1991). Monoclonal antibodies to reovirus reveal structure/function relationships between capsid proteins and genetics of susceptibility to antibody action. J. Virol..

[26] Jabre R., Sandekian V., Lemay G. (2013). Amino acid substitutions in σ1 and μ1 outer capsid proteins are selected during mammalian reovirus adaptation to Vero cells. Virus Res..

[27] Sandekian V., Lemay G. (2015). Amino acids substitutions in σ1 and μ1 outer capsid proteins of a Vero cell-adapted mammalian orthoreovirus are required for optimal virus binding and disassembly. Virus Res..

[28] Lanoie D., Côté S., Degeorges E., Lemay G. (2019). A single mutation in the mammalian orthoreovirus S1 gene is responsible for increased interferon sensitivity in a virus mutant selected in Vero cells. Virology.

[29] Szabo A., Perou C.M., Karaca M., Perreard L., Palais R., Quackenbush J.F., Bernard P.S. (2004). Statistical modeling for selecting housekeeper genes. Genome Biol..

[30] Boudreault S., Durand M., Martineau C.-A., Perreault J.-P., Lemay G., Bisaillon M. (2022). Reovirus μ2 protein modulates host cell alternative splicing by reducing protein levels of U5 snRNP core components. Nucleic Acids Res..

[31] Boudreault S., Durand M., Perreault J.-P., Lemay G., Bisaillon M. (2022). Novel roles of U5 snRNP core components in the interferon response and necroptosis during virus-host interactions. Viruses.

[32] Boudreault S., Martenon-Brodeur C., Caron M., Garant J.-M., Tremblay M.-P., Armero V.E.S., Durand M., Lapointe E., Thibault P., Tremblay-Létourneau M. (2016). Global profiling of the cellular alternative rna splicing landscape during virus-host interactions. PLoS ONE.

[33] Mohamed A., Konda P., Eaton H.E., Gujar S., Smiley J.R., Shmulevitz M. (2020). Closely related reovirus lab strains induce opposite expression of RIG-I/IFN-dependent versus -independent host genes, via mechanisms of slow replication versus polymorphisms in dsRNA binding σ3 respectively. PLoS Pathog..

[34] García-Sastre A. (2017). Ten strategies of interferon evasion by viruses. Cell Host Microbe.

[35] Lanoie D., Boudreault S., Bisaillon M., Lemay G. (2019). How many mammalian reovirus proteins are involved in the control of the interferon response?. Pathogens.

[36] DeBiasi R.L., Clarke P., Meintzer S., Jotte R., Kleinschmidt-Demasters B.K., Johnson G.L., Tyler K.L. (2003). Reovirus-induced alteration in expression of apoptosis and DNA repair genes with potential roles in viral pathogenesis. J. Virol..

[37] Guo L., Smith J.A., Abelson M., Vlasova-St Louis I., Schiff L.A., Bohjanen P.R. (2018). Reovirus infection induces stabilization and up-regulation of cellular transcripts that encode regulators of TGF-β signaling. PLoS ONE.

[38] O’Donnell S.M., Holm G.H., Pierce J.M., Tian B., Watson M.J., Chari R.S., Ballard D.W., Brasier A.R., Dermody T.S. (2006). Identification of an NF-kappaB-dependent gene network in cells infected by mammalian reovirus. J. Virol..

[39] Poggioli G.J., DeBiasi R.L., Bickel R., Jotte R., Spalding A., Johnson G.L., Tyler K.L. (2002). Reovirus-induced alterations in gene expression related to cell cycle regulation. J Virol.

[40] Smith J.A., Schmechel S.C., Raghavan A., Abelson M., Reilly C., Katze M.G., Kaufman R.J., Bohjanen P.R., Schiff L.A. (2006). Reovirus induces and benefits from an integrated cellular stress response. J. Virol..

[41] Henderson D.R., Joklik W.K. (1978). The mechanism of interferon induction by UV-irradiated reovirus. Virology.

[42] Abad A.T., Danthi P. (2020). Recognition of reovirus rnas by the innate immune system. Viruses.

[43] Abad A.T., Danthi P., López S. (2022). Early events in reovirus infection influence induction of innate immune response. J. Virol..

[44] Berger A.K., Hiller B.E., Thete D., Snyder A.J., Perez E., Upton J.W., Danthi P. (2017). Viral RNA at two stages of reovirus infection is required for the induction of necroptosis. J. Virol..

[45] Stanifer M.L., Rippert A., Kazakov A., Willemsen J., Bucher D., Bender S., Bartenschlager R., Binder M., Boulant S. (2016). Reovirus intermediate subviral particles constitute a strategy to infect intestinal epithelial cells by exploiting TGF-β dependent pro-survival signaling. Cell. Microbiol..

[46] Stuart J.D., Holm G.H., Boehme K.W. (2018). Differential delivery of genomic double-stranded RNA causes reovirus strain-specific differences in interferon regulatory factor 3 activation. J. Virol..

[47] Kniert J., Lin Q.F., Shmulevitz M. (2021). Captivating perplexities of spinareovirinae 5’ RNA caps. Viruses.

[48] Lemay G. (2018). Synthesis and translation of viral mrna in reovirus-infected cells: Progress and remaining questions. Viruses.

[49] Guo Y., Hinchman M.M., Lewandrowski M., Cross S.T., Sutherland D.M., Welsh O.L., Dermody T.S., Parker J.S.L. (2021). The multi-functional reovirus σ3 protein is a virulence factor that suppresses stress granule formation and is associated with myocardial injury. PLoS Pathog..

[50] Pan M., Alvarez-Cabrera A.L., Kang J.S., Wang L., Fan C., Zhou Z.H. (2021). Asymmetric reconstruction of mammalian reovirus reveals interactions among rna, transcriptional factor µ2 and capsid proteins. Nat. Commun..

[51] Irvin S.C., Zurney J., Ooms L.S., Chappell J.D., Dermody T.S., Sherry B. (2012). A single-amino-acid polymorphism in reovirus protein μ2 determines repression of interferon signaling and modulates myocarditis. J. Virol..

[52] Parker J.S., Broering T.J., Kim J., Higgins D.E., Nibert M.L. (2002). Reovirus core protein mu2 determines the filamentous morphology of viral inclusion bodies by interacting with and stabilizing microtubules. J. Virol..

[53] Miller C.L., Parker J.S., Dinoso J.B., Piggott C.D., Perron M.J., Nibert M.L. (2004). Increased ubiquitination and other covariant phenotypes attributed to a strain- and temperature-dependent defect of reovirus core protein mu2. J. Virol..

[54] Bisaillon M., Lemay G. (1997). Characterization of the reovirus λ1 protein RNA 5′-triphosphatase activity. J. Biol. Chem..

[55] Kim J., Parker J.S.L., Murray K.E., Nibert M.L. (2004). Nucleoside and RNA triphosphatase activities of orthoreovirus transcriptase cofactor μ2. J. Biol. Chem..

[56] Gummersheimer S.L., Danthi P. (2020). Reovirus core proteins λ1 and σ2 promote stability of disassembly intermediates and influence early replication events. J. Virol..

[57] Ooms L.S., Jerome W.G., Dermody T.S., Chappell J.D. (2012). Reovirus replication protein μ2 influences cell tropism by promoting particle assembly within viral inclusions. J. Virol..

[58] Eaton H.E., Kobayashi T., Dermody T.S., Johnston R.N., Jais P.H., Shmulevitz M. (2017). African swine fever virus np868r capping enzyme promotes reovirus rescue during reverse genetics by promoting reovirus protein expression, virion assembly, and RNA incorporation into infectious virions. J. Virol..

[59] Sherry B., Torres J., Blum M.A. (1998). Reovirus induction of and sensitivity to beta interferon in cardiac myocyte cultures correlate with induction of myocarditis and are determined by viral core proteins. J. Virol..

